# One Week Prevalence and Incidence of Diarrhea: Baseline Status of Cluster Randomised Controlled Trial of Nano Maji Filter System in Geita, Tanzania

**DOI:** 10.24248/eahrj.v6i1.684

**Published:** 2022-07

**Authors:** Kijakazi O Mashoto, Justin J Omolo, Paul E Kazyoba

**Affiliations:** a Department of Innovation, Commercialization and Technology Transfer, National Institute for Medical Research; b Mabibo Traditional Medicine Center, National Institute for Medical Research; c Directorate of Research Coordination and Promotion, National Institute for Medical Research

## Abstract

**Background:**

We have developed Nano Maji (NMM) filter system for water treatment which is currently being evaluated in a definitive cluster randomised controlled trial.

**Objectives:**

This paper descriptively presents the baseline status of one-week incidence and prevelence of diarrhoea water, sanitation and hygiene and their determinants.

**Methods:**

Recruited households in the three villages of Geita district were allocated to either intervention (NNM filter system) or control (usual practice). The primary outcome of the trial is to reduce episodes of diarrhoea at 6 months post-randomisation. Secondary outcomes are to improve water, sanitation and hygiene (WASH) status. Although households were sampled, individuals living in the selected households are used as unit of analysis for estimation of prevalence and incidence of diarrhea.

**Results:**

A total of 1,281 individuals (1,070 above 5 years and 211 under the age of 5 year children) lived in 186 households (7 individuals per household). The reported one-week prevalence and incidence of diarrhea was 10.8% and 8.4% respectively. Children under the age of five years had high incidence (22.7%) of diarrhea than individuals aged 5 years and above (5.6%). Among under five children, boys had high incidence and prevalence of diarrhea than girls. Individuals with diarrhea were likely to live in poor household, not using safe water and toilet. Over 70% of households had unacceptable latrines 135 (72.7%) and poor water situation 138 (74.3%) in terms of practice of storing, treating and drawing water from storage container. Majority of respondents had limited knowledge on handwashing and rarely used soap when washing hands.

**Conclusion:**

Substantial proportion of individuals living in project areas are affected by diarrhea. Children below the age of five years are more affected than individuals aged five years and above. The baseline findings are representative of local status of WASH, and reflects the prevailing poor water, sanitation and hygiene status in rural areas of Tanzania. Trial approval number NIMR/HQ/R.8a/Vol. IX/3045.

## BACKGROUND

Although simple and cost effective interventions to prevent and treat diarrhoea are available, childhood diarrheal disease is still among the leading causes of mortality and morbidity.^1,2^ In 2016, mortality due to diarrhoea was 22.4 deaths (16.8–32.0) per 100,000 globally. Higher rates were recorded among children younger than 5 years (70.6 deaths per 100,000) and among individuals older than 70 years (171.7 deaths per 100,000. In the same year, diarrhoea contributed 8.9% of all deaths in children younger than 5 years. The estimated episodes of diarrhoea among children younger than 5 years were 1,105,406,865 which translated into 1.75 episodes (1.52–2.02) per child.^3^ The highest burden of diarrheal related mortality is carried by developing countries with nearly four fifths of all under-five mortality occurring in Sub-Saharan Africa and south Asia.^4,5^

Substantial progress has been made globally in reducing the burden of diarrhoeal diseases, driven by decreases in several primary risk factors. However, this reduction is not equally distributed across locations. Evidence shows that diarrhoeal diseases disproportionately affect communities with poor access to health care, safe water, and sanitation, and low-income or marginalised populations.^6^ In Tanzania, diarrheal diseases are major contributors to under-five mortality. According to the 2016 Tanzania Demographic and Health Survey report, 12% of under-five children had a diarrheal episode in the 2 weeks prior to the survey.^7^ The 2016 Tanzania Demographic and Health Survey reported that while there was no notable difference in diarrhoea prevalence among children by source of household drinking water and toilet facility, the prevalence varied with mother's le vel of education and household wealth.^8^

Several other studies conducted in Tanzania and elsewhere have also reported similar and varying prevalence and incidences of diarrhoea among under-five children.^9-12^

The areas where artisanal mining activities are taking place are characterised with high population and poor provisions of water, hygiene and sanitation services. As a result, these areas have a high burden of water borne diseases. Previous study conducted in Geita region, reported two weeks' diarrhoea prevalence of 28.3% among under-five children and 15.5% among their mothers/caretakers.^12^ It should be noted that there is paucity of data regarding the magnitude of diarrheal disease burden among individuals aged five years and above living in mining areas of Geita. This study was conducted with the aim to determine prevalence and incidence of diarrhoea among individuals living in randomly selected households in Busolwa and Mgusu wards in Geita region, Tanzania. The results of this study will be used to increase knowledge on the burden of diarrhoea in the selected villages which are in proximity to mining areas, and monitor and evaluate the effect of Nano Maji Filter System (NNM) intervention on health outcomes.

### Intervention with Nano Maji Filter System (NNM)

NNM is a water treatment system designed to remove heavy metals and microbial contamination in water. In this survey, we undertook a randomised controlled field trial to evaluate the effectiveness of NNM system to remove heavy metals and microbial contaminants in domestic water. The primary outcome of the trial was to reduce episodes of diarrhoea at 6months post-randomisation. Secondary outcomes were to improve water, sanitation and hygiene (WASH) status. The trial was implemented in 3 villages of Geita where 64 households were selected to receive the intervention (NNM system and WASH education) and 128 households were put in control arm (continue with usual practice). At baseline, information on episodes of diarrhoea in the past one week prior to ther survey was collected for all participating household members. Weekly visitations were made to participating households for a period of 3 months. Individuals in these households were the unit of analysis for estimation of prevalence and incidences of diarrhoea.

## METHODS

### Study Site

Most mines are located in remote areas and they lack adequate sanitation, thus making individuals living in these areas vulnerable to waterborne diseases.^13^ For this reason, villages located in mine areas in Busolwa and Mgusu wards were selected. The selected wards are situated in Geita which is one of Tanzania's 31 administrative regions. It comprises of 5 districts and 6 councils, one of which (Geita) is a town council. The districts are divided into divisions that are further subdivided into wards, villages, and sub-villages (hamlets). In total, the region is made up of 21 divisions, 122 wards, 474 villages, 65 streets, and 2219 sub-villages or hamlets.

The region covers an area of 20,054 square kilometres (7,743 square meters), bordered by Lake Victoria to the east, and home to Tanzania's largest gold mining industries. The Geita Gold Mine is the largest mining operation located within Geita Region, 4 km west of the town of Geita. Other major industries in the region are agriculture and fishing. Geita Region has moderate temperatures of between 220 degrees Centigrade and 300 degrees Centigrade with average rainfall ranging from 900 mm to 1200 mm per annum. Rainfall is fairly evenly distributed with short rains from September to December followed by a dry spell from January to February before long and heavy rains set in from March till the end of May. From June to September the region is subjected to dry season. During the hot season, humidity is 35%, and rises up to 60% during the rainy season. Water supply in Geita Region is satisfactory with Lake Victoria serving as the main water source in the area. Other water sources include rivers, streams, shallow wells, bore holes, rain water harvesting and springs. The demand for water in this region is driven by human and livestock population. In 2018, demand for water in Geita Region was reported at 53,149 cubic meters (m^3^) against availability of 27,637.5 m^3^, which is 53% of the region's total demand. Water supply in urban areas (i.e. Geita Town, Chato Town and Bukombe Town) was at 41%.^14^

### Study Design

A cross sectional study was conducted to generate baseline data which was used to monitor and evaluate the performance and effect of NNM water filter system in removing heavy metals and microbial contaminations from domestic water.

### Study Population

Study population include all individuals residing in selected households for a randomised field trial to evaluate the effectiveness of NNM water filter system in removing heavy metals and microbial contaminants from domestic water.

### Sample Size

Sample size was calculated using StatCalc - Epi Info™ 7 which is an online sample size calculator. The assumptions used in sample size calculation were; 80% study power, 95% Confidence Intervals (CI), and risk ratio of 1.84 for exposed group (control arm), the calculated Kelsey sample size was 194 households. (Control group 129 and experiment group 65). Information on the occurrence of diarrhoea was collected from all individuals residing in each of the selected 194 households involved in the trial.

### Sampling procedures

While wards and villages were purposeful selected, households were systematically selected from the list of all households with at least one child under the age of 5 years obtained from the 3 Village Executive Officers (VEOs). For each village, the total number of required households was divided by the total number of households in the sampling frame to get a number which was used to select the first and the next household. A total of 3 villages were selected from 2 wards (two villages from Mgusu ward and one village from Busolwa ward). From each village, 68 households were selected.

### Inclusion Criteria

Villages were selected based on availability of water sources which are used by at east 80% of the population, availability of health facility and proximity to the mining areas. Selection of household was based on availability of at least one child under the age of five years living in the household.

### Exclusion Criteria

The village was excluded if it lacks health facility and a common water source which is used by at least 80% of village population, and not located close to the mining areas. Households without a child under the age of five years were also excluded from the sampling list.

### Ethical Considerations

The study was reviewed by the National Health Research Review Committee (NatHREC) and granted certificate of approval with number NIMR/HQ/R.8a/Vol. IX/3045. Permission to conduct the study was obtained from local government authorities in Geita at all levels (region, district, ward and village).

The study used written informed consent form (ICF) for adults (18+ years). The form contained a general description of the study (e.g., procedures, lengths, goals and objectives) as well as a statement on risks and benefits associated with the study and an explanation of key rules such as confidentiality and voluntary participation. Contact information for the research team were also provided in case participant wished to reach out for further clarifications.

Verbal permission was sought for from household members aged 10 to 17 years to allow respondents share their information on diarrhoea episodes. Respondents and household members were assured that their decision not to participate in the project or to be interviewed or not to answer certain questions or to stop the participation at any stage of the study was ok and would not jeopardize their rights to access medical care or other social services.

### Data Collection and Analysis

The study adopted data collection tools which were used to estimate one-week incidence and prevalence of diarrhoea among children under the age of five years in Mkuranga distriict.^10^ The adopted data collection tools were translated into Swahili. Translation was done by native qualified translators and were back translated by a different person who had not seen the English questionnaire to see if the tools had the same meaning. Any inconsistencies in the translation were addressed. The approved translated tools were scripted into a table format. The tools were administered by Research Assistants who underwent a 3-day training prior to the data collection exercise.

Of the 486 eligible households in the intervention arm (Busolwa), 65 households were sampled and consented to participate in the project. In control arm, 995 households were eligible but the study sampled only 121 households, the response rate was 100%. Interviews with heads of households, observations and census of household members were the methods of data collection employed by this study. In the event that the head of the household was not present, any other member of household aged 15 years and above was interviewed. The tool used in the previous study^10^ collected information on respondents' hygiene practice and knowledge, water treatment practice and household sanitation, captured information on occurrence of episodes of diarrhoea among children below the age of five years and adults residing in visited households. Encouraged by other researchers,^14,15^ this study used the World Health Organization's definition of diarrhoea to assess episodes of diarrhoea.^16^

Knowledge on hygiene was assessed by 9 items which had yes and no responses. Respondents were asked when is the critical time to hands. The final score was obtained by summing the items and then dichotomised into less and comprehensive hygiene knowledge.

Sanitation score was constructed by summation of five main observations around the household. These included presence or absence of animal or human faeces, trash and pit for dumping trashes around the homestead and utensil drying rack. Presence was coded 1 and absence 2. The sanitation score was constructed by adding all the five items and then dichotomised responses into poor sanitation and good sanitation.

Water situation was assessed by rating households' main water source, the practice of storage and treatment of drinking water and the practice of drawing water from the storage container. First, the water sources were rated as safe (coded 1 and included borehole) or not safe (coded 2 included shallow well, river, pond, lake, canal and stream). Frequency of each water treatment method was rated as always (coded 1), sometimes (coded 2) rarely (coded 3) and never (coded 4). After summation of frequencies of all the water treatment methods, the score was dichotomised into adequate water treatment practice (coded 1) and inadequate water treatment practice (coded 2). Water storage practice was coded 1 if the household use gallon, 2 if uses pot and 3 if the household uses bucket with no cock. However, in the analysis code 2 and 3 were combined and ended up with good water storage practice (code 1) and poor water storage practice (code 2). The practice of drawing water from the storage container was assessed by asking respondents to select the appropriate option which included a cup used only for drawing water (coded as 1), a cup used for drawing water and sometimes for drinking (coded as 2) and a cup used for both drawing and drinking (coded as 3). In the analysis, option 2 and 3 were merged and therefore this item remained with two options 1 being good water drawing practice and 2, poor water drawing practice. To get a composite index for water situation, the four items were summed up (water source safety, and water treatment, storage and drawing practices) with high score indicating poor water situation. The study used mean score as the cutoff point for dichotomising the resulted scores into good water situation coded as 1 and poor water situation coded as 0.

Occurrence of diarrhoea was assessed by asking respondent if any member of the household had diarrhoea in the previous 7 days. If the answer is yes, the respondent was asked on which days member of the household had diarrhoea and how frequently a person passed out loose or watery stool within 24 hours. In addition, respondents were asked how long the diarrhoea lasted (number of days with diarrhoea).

The approach used by Mashoto et al^10^ to assess wealth index was adopted. Household assets such as house, toilet, land, radio, motorcycle, bicycle, car, phone, cow, pig, goat, sheep, donkey, chicken and duck were recorded as 1 “available and functioning” and 0 not available or available not functioning. Use of sources of energy for cooking i.e. electricity, kerosene oil, fire woods, gas and solar were recorded as 1 “yes” or 0 “no”. Assets and sources of energy for cooking were analysed using principal components analysis (PCA). The first component resulting from the analysis was used to categorise households into 4 approximate quintiles of wealth ranging from the 1st poorest to the least poor 4th quintile.

Data was analysed using IBM SPSS Statistics for Windows version 19.0 (IBM Corp, Armonk, NY, USA). Continuous variables were summarized into mean and standard deviation while categorical ones were summarised into proportions. Chi-square test and multi nominal logistic regression were applied to determine factors influencing knowledge of causes of diarrhoea. We calculated the prevalence of diarrhoea as the percentage of individuals suffering from diarrhoea assessed during a 7-day period.

### Data Management

The data was entered automatically using the data collection software during the fieldwork. There were limited number of paper versions of the survey available to the interviewers in case of any problem with electronic devices; paper-based data were entered into the electronic devices at the end of the day when there was a connection. All data were checked on a daily basis via random verification checks by the field monitoring team. The data was checked for duplicated entries, outliers, spurious codes. Additional cross-checking was done to triangulate and eliminate entries with mutually exclusive answers on a range of questions. The team used call-backs to verify spurious or suspicious answers with the respondents. Where not possible, entries containing such answers were removed.

Two data sets were maintained – one with the respondent personal and contact information and the other one with the actual responses to the survey questions including diarrhoea information for all household members. Only the senior research team had access to the dataset with the respondent identifiable data.

## RESULTS

**FIGURE 1:**
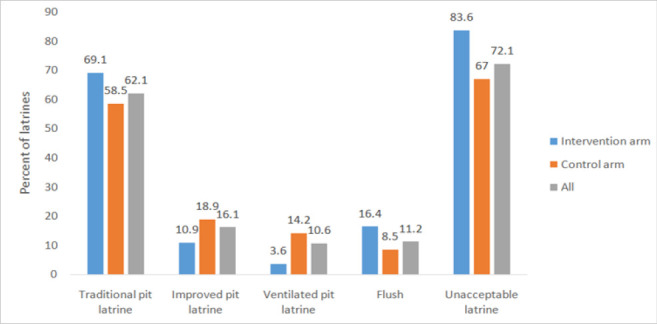
Types and Status of Households' Latrines

Out of 194 households, interviews were possible in 186 households (response rate 95.9%). A total of 1,281 individuals (1,070 above 5 years and 211 under the age of 5 year children) lived in 186 households (on average household had 7 individuals). Almost 60% of the respondents were married male peasants who attained primary school education level. The majority of households (62.1%) use traditional pit latrine (Figure 1). Over 70% of the households have poor water situation in terms of water storage, treatment and practice of drawing water from the storage container. In 76.8% of the visited households, drinking water is not treated. Significantly high proportion of households in the intervention arm do not treat drinking water (Table 2).

**TABLE 1:** Response Rate and Respondents' Profile

	Intervention arm (n = 65)	Control arm (n = 121)	All (N = 186)
Response rate	100%	94.5%	96.9%
Mean Age (SD)	40.9 (10.7)	44.2 (11.5)	43.0 (11.3)
**Sex**			
Males	58 (89.2)	98 (81.0)	156 (83.9)
Females	7 (10.8)	23 (19.0)	30 (16.1)
**Position in houses**			
Head of house	25 (38.5)	88 (72.7)^**^	113 (60.8)
Spouse/Partner	30 (46.1)	24 (19.8)	54 (29.0)
Child/Relative	10 (15.4)	9 (7.4)	19 (10.2)
**Marital status**			
Married	39 (60.0)	71 (58.7)	110 (59.1)
Cohabiting	21 (32.3)	29 (24.0)	50 (26.9)
Other	5 (7.7)	21 (17.4)	26 (14.0)
**Went to school**			
Yes	63 (96.9)	100 (82.6)	163 (87.6)
No	2 (3.1)	21 (17.4)^**^	23 (12.4)
**Education level**			
Primary school	56 (88.9)	82 (82.0)	138 (84.7)
Secondary school	7 (11.1)	18 (18.0)	25 (15.3)
**Occupation**			
Peasant/livestock keepers	45 (69.2)	65 (53.7)	110 (59.1)
Small business	11 (16.9)	28 (23.1)	39 (21.0)
Employed	0 (0.0)	5 (4.1)	5 (2.7)
Small scale mining	9 (13.8)	23 (19.0)	32 (17.2)
**Wealth Index**			
1st least poor	17 (26.2)	37 (30.6)	54 (29.0)
2nd poor	23 (35.4)^*^	24 (19.8)	47 (25.3)
3rd poor	16 (24.6)	25 (20.7)	41 (22.0)
4th poorest	9 (13.8)	35 (28.9)^*^	44 (23.7)

* P<0.05;

** P<0.001

**TABLE 2:** Respondents' Wash Hand Practice, and Households' Water and Hygiene Status

	Intervention arm (N= 65)	Control arm (N = 121)	All (N = 186)
Household do not treat drinking water	80 (86.0)^*^	82 (69.5)	162 (76.8)
Household poor water storage	29 (31.2)	42 (35.6)	71 (33.6)
Household poor water situation	71 (76.3)	93 (78.8)	164 (77.7)
Respondents wash hands at critical moments	30 (32.3)	48 (40.7)	78 (37.0)
Respondent always use running water to wash hands	40 (43.0)	39 (33.1)	79 (37.4)
Respondent always use soap to wash hands	15 (16.1)	19 (16.1)	34 (16.1)
Always respondent use toilet	80 (86.0)	108 (91.5)	188 (89.1)
Knowledge on soap kill germs	60 (92.3)	108 (89.3)	168 (90.3)

* P<0.05

Slightly over one third of the interviewed individuals reported to always use running water to wash their hands at critical moments. However, consistence use of soap when washing hand was affirmed by 16% of the - respondents (Table 2). In few of the visited households, visible human and animal stools were spotted on the surroundings, and about a third of households had pit for dumping trash. The total sanitation score being lower in the control arm than in the intervention arm (Table 3).

**TABLE 3:** Household Hygiene and Sanitation

	Intervention (n = 65)	Control arm (n = 121)	All (N = 186)
Human stool near house	3 (4.6)	5 (4.1)	8 (4.3)
Animal stool near house	16 (24.6)	16 (31.2)	32 (17.2)
Trash near house	32 (49.2)^*^	42 (34.7)	74 (39.8)
Pit for dumping trash	17 (26.2)	34 (28.1)	51 (27.4)
Utensil rack	11 (16.9)	38 (31.4)^*^	49 (26.3)
**Household Sanitation Score**			
Good	37 (56.9)	85 (70.2)	122 (65.6)
Poor	28 (43.1)^*^	36 (29.8)	64 (34.4)

* P<0.05

The reported one-week prevalence and incidence of diarrhoea among 1,281 individuals living in the surveyed 186 households was 10.8% and 8.4% respectively. Individuals living in households allocated for intervention and control were equally affected with diarrhoea (Table 4). Compared to adults (5.6%), children under the age of five years had high incidence (22.7%) of diarrhoea. Within group variation existed with boys under the age five years being more affected than girls (Table 5). Table 6 depict results of multivariate analysis which indicate that individuals with diarrhoea were more likely to live in the households which occasionally use soap for hand washing (OR 6.3; 95% CI, 1.9 to 21.4) and less likely to use toilets (OR 0.1; 95% CI,0.03 to 0.4).

**TABLE 4:** One-Week Incidence and Prevalence of Diarrhoea among Individuals Living in Households Assigned to Intervention and Control Arm

	Intervention (n = 524)	Control (n = 757)	All (N = 1281)
Diarrhoea events	48	60	108
Incidence (%)	9.2	7.9	8.4
Prevalence (%)	62 (11.8)	76 (10.0)	138 (10.8)

**TABLE 5:** One-Week Incidence and Prevalence of Diarrhoea among Individuals Living in the Surveyed Households by Age Group (N = 1281)

	Incidence		Prevalence	
	<5 years	≥5 years	<5 years	≥5 years
All	22.7^**^	5.6	29 (13.7)	109 (9.5)
Sex				
Males	43.6^**^	0.7	18 (17.8)^*^	51 (9.7)
Females	20.0	9.0^**^	11 (10.0)	58 (10.7)
Group				
Intervention	20.4	3.7	15 (16.1)	47 (10.9)
Control	24.6	6.9	14 (11.9)	62 (9.7)
Wealth index				
Less poor quintile	15.4	1.8	9 (13.8)	24 (7.4)
Least poor quintile	22.8	9.8^*^	9 (13.8)	36 (12.2)
Poorer quintile	6.4	2.7	4 (8.5)	22 (8.6)
Poorest quintile	52.4^**^	9.3^*^	7 (16.7)^*^	27 (14.0)^*^

* P < 0.05

** P > 0.001

**TABLE 6:** Factors Associated with Diarrhoea (Those with Diarrhoea)

R2 = 0.229	OR	95% Cl	P value
**Sex**			
Females	0.6	0.2–1.9	0.402
Males	1		
**Wealth Index:**			
Less poor quintile	0.8	0.6–4.4	0.842
Least poor quintile	1.3	0.3–0.5	0.691
Poorer quintile	0.5	0.1–2.3	0.374
Poorest quintile	1		
**Intervention group**			
Yes	1.6	0.6–4.3	0.307
No	1		
**Household water situation**			
Good	1.1	0.3–5.0	0.858
Poor	1		
**Defecation place**			
Toilet	0.1	0.03–0.4	0.002
Open	1		
**Wash hands at critical moments**			
Yes	1.1	0.4–3.2	0.811
No	1		
**Use running water to wash hands**			
Yes	0.31	0.1 – 1.1	0.070
No	1		
**Soap use to wash hands**			
Occasionally	6.3	1.9–21.4	0.003
Always	1		

## DISCUSSION

Provision of access to safe water, sanitation and hygiene are the most impactful interventions for reduction of diarrhoea. Poor water situation coupled with inadequate household sanitation and limited hygiene practice exposes both adults and under-five children to diarrhoea. One in four households are at risk of consuming contaminated drinking water, increasing their susceptibility to episodes of diarrhoea, particularly in under-five children who had high diarrheal incidence and prevalence than adults. The incidence and prevalence of diarrhoea among children under the age of five years reported in this study are higher than what have been reported in Mkuranga district^10^ but lower than the incidence revealed by systematic review of diarrheal incidence in low and middle income countries^16^ and diarrhoea prevalence reported in rural area of Nigeria.^17^ However, the Nigeria study assessed two-weeks diarrhoea prevalence while the current and the previous studies conducted in Tanzania assessed one-week prevalence.

Previous studies have demonstrated that low socioeconomic status, lack of education, poor water storage practices, use of unsafe water, poor sanitation and overcrowding in terms of high number of children under the age five years living in a household are risk factors for diarrhoea.^10,18-21^ In the present study, individuals belonging to the poorest wealth quintile were more likely to have diarrhoea but the significant association disappeared in the multivariate analysis. Our study adds to the body of knowledge that use of toilets and soap when washing hand is critical for prevention of diarrhoea.

Results of our study revealed that children under the age of five years have high incidence and prevalence of diarrhoea than individuals aged 5 years and above. However, in the first stage of multivariate analysis, the significant association between prevalence of diarrhoea and age disappeared. Thus, in order to prevent or reduce episodes of diarrhoea among children and adults, it is important to implement water, sanitation and hygiene interventions at individual, household and community levels.

The observed differences in the water treatment practice and sanitation status between households in intervention and control arm disappeared in the multivariate analysis, and thus it is unlikely such slight difference will have any impact on outcome of the trial.

### Study Limitations

It should be noted that the prevalence of diarrhoea is seasonal and subject to the respondent's reporting of the episode of diarrhoea. We collected data during dry season and hence the reported prevalence and relied on information provided by respondent on behalf of him/herself and other members of the household. Thus it is possible that the study under estimated the incidence and prevalence of diarrhoea due to both seasonal variations and recall bias or respondent not having correct information on diarrhoea episodes for all of his/her household members because they did not disclose the information to the respondent.

## CONCLUSION

Substantial proportion of individuals living in the study area are affected by diarrhoea. Children below the age of five years are more affected than individuals aged five years and above. However, it is likely that the study under estimated the incidence and prevalence of diarrhoea among individuals aged 5 years and above due to the biases described in the study limitations section. Nevertheless, the study provides baseline data for monitoring and evaluating the health outcome of NMM water filter system.
